# Editorial: Functional acquired hypogonadotropic hypogonadism in males

**DOI:** 10.3389/fendo.2024.1364634

**Published:** 2024-01-31

**Authors:** Biagio Cangiano, Marco Bonomi, Richard Quinton

**Affiliations:** ^1^ Department of Medical Biotechnology and Translational Medicine, University of Milan, Milan, Italy; ^2^ Department of Endocrine and Metabolic Medicine, IRCCS Istituto Auxologico Italiano, Milan, Italy; ^3^ Department of Endocrinology, Diabetes & Metabolism, Newcastle-upon-Tyne Hospitals, Newcastle-upon-Tyne, United Kingdom; ^4^ Translational & Clinical Research Institute, University of Newcastle-upon-Tyne, Newcastle-upon-Tyne, United Kingdom; ^5^ Department of Metabolism, Digestion & Reproduction, Imperial College London, London, United Kingdom

**Keywords:** functional hypogonadotropic hypogonadism, testosterone, obesity, metabolic syndrome, relative energy deficit, genetics, chronic disease

Adult-onset isolated hypogonadotropic hypogonadism (HH) is an heterogeneous condition associated with signs and symptoms that can significantly impact on patients’ quality of life through gynecomastia, osteoporosis, anaemia, sexual dysfunction and impaired fertility, and may also be linked with other chronic conditions such as osteoporosis, depression and cardiometabolic disease.

HH is classically divided into organic (due to structural or genetic damage) and “functional” (FHH - related to comorbidities, aging and/or medication) forms, with the latter being particularly prevalent among patients with chronic diseases. These conditions differ for the potential reversibility of the disease and the pathogenic mechanisms, but also (as recent data here reviewed by Grande et al.) in relation to different effects on biochemical pathways, including glucose metabolism and ketogenesis. These data support the view that this form of hypogonadism should be considered and addressed not only in relation to low testosterone levels, but also for its ancillary features related to the underlying disease (1).

Although relative FHH due to energy deficit is now increasingly recognized in males (2) as well as females, that associated with diabesity is the by far most prevalent form. Even if clinical trials of testosterone treatment in FHH have reported improvements in lipid profile, body composition, insulin sensitivity and glycemic control in dysmetabolic patients, this therapy can be associated with unacceptable degrees of erythrocytosis (3), particularly where there is a concomitant obstructive sleep apnoea. Indeed, data from the T-trials suggest that the greatest functional improvements may accrue to men with baseline anaemia (4).

There is still no consensus as to whether any form of FHH should be treated with testosterone, or other hormonal therapies, such as aromatase inhibitors (AI) or selective estrogen receptor modulators (SERM) as part of routine clinical care. This is because of the potential for complete reversibility of the condition, including restoration of fertility (which cannot be achieved by testosterone treatment) with lifestyle measures alone. Moreover, the improvements in fertility and sexuality observed with these AI and SERM may be marginal (5, 6) in addition to a potential negative impact of AI on bone density (6).

The pathogenetic mechanism of FHH is also a matter of debate, and there are different hypotheses. In dysmetabolic forms, for example, estrogen excess due to adipose aromatization was initially thought to cause hypogonadism through negative feedback. However, despite the biochemical responsiveness of the male hypothalamo-pituitary-testicular axis in obesity and chronic disease to blockade of estrogen secretion or action (5, 6), the impairment of gonadotropin secretion in FHH is more convincingly attributed to systemic inflammation, or a reduced neuronal sensitivity to insulin and/or leptin. Finally, it has been shown that, in adult-onset HH, there is enrichment of rare variants in the genes related to congenital HH, suggesting an individual predisposition (7). However these mechanisms cannot be robustly extended to other forms of FHH (8), including prolonged therapy with opioids, corticosteroids and dopamine-antagonists, abuse of anabolic-androgenic steroids, relative energy deficit due to low fat mass from excessive physical endurance training, as well as hypogonadism due to various chronic diseases (9, 10). Spaziani et al. and colleagues, explore the many aspects of this elusive definition and the current knowledge about functional HH in a mini review of this Research Topic.

In fact, all these conditions are often considered as a whole, within the wider category of FHH, which intrinsically limits the understanding of the peculiarities in both the pathogenesis and clinical outcomes of what are clearly different diseases, that until now lack the due insight. Also, many other conditions associated with low testosterone levels are variably included or excluded from this great pot, such as depression and mood disorders, as well as HIV related hypogonadism (here reviewed respectively by Indirli et al. and De Vincentis and Rochira adding complexity to the matter.

In the age of a medicine aimed to restore not only patients’ life expectancy but also their quality of life, it is important for future research to focus on the clinical consequences of each functional form of hypogonadism, and when and how to treat them.

## Author contributions

BC: Writing – original draft, Writing – review & editing. MB: Writing – review & editing. RQ: Writing – review & editing.
